# Supplementary material for: Content-rich biological network constructed by mining PubMed abstracts
Source: BMC Bioinformatics. 2004 Oct 8;5:147. doi: 10.1186/1471-2105-5-147 (PMC528731; doi:10.1186/1471-2105-5-147)
Supplement: Additional File 2 — The original results of the above study (non-essential files are deleted to keep the file size under the limit set by BMC bioinformatics). [file 1471-2105-5-147-S2.bz2 › chilibotAdditionalFile2/dip10/94ID9178909E401/html/CBL_CRK.html]

 


 **CBL** and **CRK** 
  
Found 57 abstracts in PubMed, retrieved 10.  
 

 What does Google say? 
 PDF only 
| .edu only 

---

**Interactive relationship** (e.g. stimulation, inhibition, etc)

**Neutral relationship**- In  **Cbl**  b T cells, the interaction between  **Crk**  L and C3G, and the activity of the small GTPase Rap1 are increased.  Ref: 12697763 J Biol Chem< MedlineTA>J Biol Chem,
- It was previously reported that  **Cbl**  b associates with  **Crk**  L in Jurkat T cells.  Ref: 12697763 J Biol Chem< MedlineTA>J Biol Chem,
- Thus,  **Cbl**  b plays a negative role in  **Crk**  L C3G mediated Rap1 and LFA 1 activation in T cells.  Ref: 12697763 J Biol Chem< MedlineTA>J Biol Chem,
- We found that  **Cbl**  b associates with, and induces ubiquitin conjugation to  **Crk**  L, which requires a functional RING finger.  Ref: 12697763 J Biol Chem< MedlineTA>J Biol Chem,
- Although the exact mechanism of interaction between these molecules has not been fully defined, it has been generally accepted that  **Crk** , ZAP 70, and p85 interact with tyrosine phosphorylated  **Cbl** , which serves as a major scaffold protein in activated T lymphocytes.  Ref: 11418612 J Biol Chem, 2001
- T cell activation induces direct binding of the  **Crk**  adapter protein to the regulatory subunit of phosphatidylinositol 3 kinase p85 via a complex mechanism involving the  **Cbl**  protein.  Ref: 11418612 J Biol Chem, 2001
- The  **Crk**  adapter proteins are assumed to play a role in T lymphocyte activation because of their induced association with tyrosine phosphorylated proteins, such as ZAP 70 and  **Cbl** , and with the phosphatidylinositol 3kinase regulatory subunit, p85, following engagement of the T cell antigen receptor.  Ref: 11418612 J Biol Chem, 2001
- The overexpression of a CAP mutant in which the SoHo domain had been deleted CAPDeltaSoHo prevented the translocation of  **Cbl**  to lipid rafts and subsequently blocked the recruitment of CrkII  [ **CRK** ]  and C3G.  Ref: 11481476 Proc Natl Acad Sci U S A, 2001
- Like c  **Cbl** ,  **Cbl**  b associates constitutively with CAP and interacts with  **Crk**  upon insulin stimulation.  Ref: 12842890 J Biol Chem< MedlineTA>J Biol Chem, 2001

**Non-interactive relationship** (e.g. studied together, co-existance, homology, etc.)

- Overexpression of the APS Y 618 F mutant in 3T3 L1 adipocytes blocked the insulin stimulated tyrosine phosphorylation of endogenous  **Cbl**  and binding to  **Crk** .  Ref: 11997497 Mol Cell Biol, 2002
- It was found in complexes with many signaling proteins, including phosphoinositol PtdIns 3 kinase EC 2.7.1.137,  **Cbl** , GRB2, p130Cas and  **Crk** .  Ref: 12135478 Eur J Biochem, 2002
- When overexpressed in these cells, wild type APS but not an APS Y 618 F mutant facilitated the tyrosine phosphorylation of coexpressed  **Cbl**  and its association with  **Crk**  upon insulin stimulation.  Ref: 11997497 Mol Cell Biol, 2002
- We demonstrate here that PKA s activation of Rap1 was mediated by the Rap1 guanine nucleotide exchange factor C3G, the adaptor  **Crk**  L, the scaffold protein  **Cbl** , and the tyrosine kinase Src.  Ref: 11804588 Mol Cell, 2002
- EphA activation also induced  **Cbl**  association with  **Crk**  L and  **Crk**  II  [ **CRK** ]  adapters, but not the related Grb2 protein.  Ref: 12794130 J Immunol, 2003
- Here we examined a regulatory role of  **Cbl**  b in  **Crk**  L C3G signaling pathway.  Ref: 12697763 J Biol Chem< MedlineTA>J Biol Chem, 2003
- Negative regulation of TCR mediated  **Crk**  L C3G signaling and cell adhesion by  **Cbl**  b.  Ref: 12697763 J Biol Chem< MedlineTA>J Biol Chem, 2003
- **Cbl**  b deficiency does not affect  **Crk**  L stability, but its association with C3G.  Ref: 12697763 J Biol Chem< MedlineTA>J Biol Chem, 2003
